# Unveiling the GA_4_-Ferulic Acid Regulatory Axis: Redox-Mediated Suberization Governs Adventitious Rooting Recalcitrance in *Pinus massoniana*

**DOI:** 10.3390/plants14213246

**Published:** 2025-10-23

**Authors:** Yin Wang, Ruiling Yao

**Affiliations:** Guangxi Forestry Research Institute, Nanning 530002, China; yinvvang@163.com

**Keywords:** gibberellin A_4_, ferulic acid, redox homeostasis, suberin, adventitious rooting, *Pinus massoniana*

## Abstract

*Pinus massoniana*, a critically important afforestation species in subtropical China, shows severe adventitious rooting recalcitrance linked to endogenous gibberellin (GA) dysregulation. Our study reveals a GA_4_-mediated regulatory network that coordinates hormonal crosstalk, redox homeostasis, and cell wall remodeling. Treatment with the GA biosynthesis inhibitor paclobutrazol (PBZ, 100 mg·L^−1^) shortened rooting time by 32.5% and increased rooting success by 79.5%. We found that PBZ redirected GA flux by upregulating GA_3_-oxidase (GA_3_OX), leading to GA_4_ accumulation. However, elevated GA_4_ levels impaired root development by triggering suberization through ferulic acid (FA)-mediated redox imbalance. Application of GA_4_ (100 mg·L^−1^) reduced caffeoyl alcohol content by 54.4% but increased FA and caffeic acid levels 2.4–3.9-fold, shifting lignin precursors toward suberin biosynthesis. FA modulated H_2_O_2_ flux in a dose-dependent manner: 200 mg·L^−1^ optimized redox homeostasis (93.7% lower H_2_O_2_ influx), whereas 1000 mg·L^−1^ suppressed mitosis. The combination of PBZ (100 mg·L^−1^) and FA (200 mg·L^−1^) synergistically enhanced rooting success by 34.4% and achieved 95.8% field survival after two years (vs. 68.5% in controls), challenging the traditional view that lignification alone limits rooting in woody plants. This work provides the first evidence that the GA_4_-FA axis controls adventitious root formation in conifers via a Reactive oxygen species (ROS)-dependent switch between suberin and lignin metabolism, offering new strategies to overcome rooting barriers. The PBZ + FA protocol enables scalable clonal propagation of recalcitrant conifers, with potential applications in molecular breeding and forest restoration.

## 1. Introduction

*Pinus massoniana*, a cornerstone species in southern China’s reforestation efforts, faces persistent challenges in clonal propagation due to its recalcitrant adventitious rooting capacity [1]. While auxin-mediated cell division and differentiation is widely recognized as a key driver of rooting [2], the role of gibberellins (GAs) remains controversial. GAs are traditionally viewed as promoters of cell division and dormancy release [3], yet emerging evidence suggests their dysregulation inhibits rooting in woody plants [4,5]. Notably, *P. massoniana* exhibits a unique biphasic rooting process: a root induction phase (0–10 days) and root expression phase (10–35 days), which complicates GA-mediated regulation [6].

Although paclobutrazol (PBZ), a GA biosynthesis inhibitor, improves rooting rates in *P. massoniana* [5], its molecular mechanisms remain unclear, specifically, (i) GA isoform specificity: PBZ reduces total GA content, but its effects on individual isoforms (e.g., GA_3_ vs. GA_4_) and their temporal dynamics are uncharacterized. (ii) Lignin-suberin interplay: PBZ enhances lignin precursor accumulation and suberization [6,7], yet their functional linkage to rooting inhibition is unresolved. (iii) Reactive oxygen species (ROS) signaling: ROS regulate cell wall remodeling during rooting [4], but their role in *P. massoniana* is unexplored. Critically, no study has integrated GA isoform dynamics, lignin/suberin metabolism, and ROS signaling to explain the molecular basis of rooting recalcitrance in conifers. Addressing these gaps is essential for developing targeted strategies to improve asexual propagation efficiency in *P. massoniana*.

This study integrates physiological, metabolic, and anatomical approaches to elucidate the regulatory mechanisms of adventitious rooting in *P. massoniana* cuttings. Key objectives include characterizing GA isoform dynamics (quantifying changes in 16 endogenous GAs during PBZ-mediated rooting inhibition), dissecting lignin-suberin crosstalk (analyzing the impact of gibberellin GA_4_ on lignin precursors and suberin deposition in rhizome tissues), and decoding ROS signaling (investigating the role of ferulic acid (FA) in mediating H_2_O_2_ homeostasis and root development). This work provides a foundation for understanding the molecular basis of rooting dysfunction in recalcitrant conifers and offers actionable insights for optimizing asexual propagation practices.

## 2. Results

### 2.1. PBZ Treatment Optimizes Rooting Kinetics

**Figure 1:**
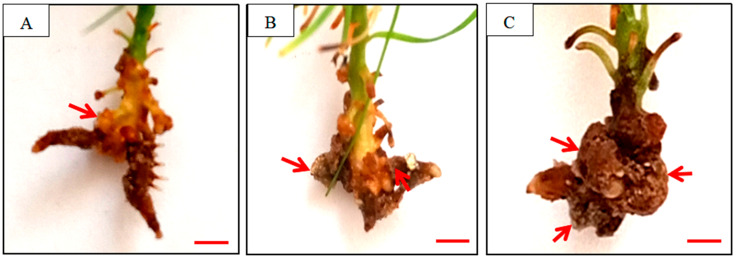
Root morphology of *Pinus massoniana* cuttings under PBZ treatment of 35 days. (**A**) 200 mg·L^−1^ NAA; (**B**) 200 mg·L^−1^ NAA + 100 mg·L^−1^ PBZ; (**C**) 200 mg·L^−1^ NAA + 200 mg·L^−1^ PBZ. Arrows show the callus tissue at the base of cuttings. Scale bars: 0.5 cm.

PBZ treatment alone had no rooting effect, whereas its combination with NAA induced improved rooting effect. Compared to the NAA control, co-application of PBZ with NAA significantly enhanced rooting efficiency (Table 1). Rooting time was reduced by 32.5% (from 45.2 ± 1.5 d to 30.5 ± 2.3 d, *p* < 0.05), and rooting percentage was increased from 58.6 ± 3.3% (Control) to 79.5 ± 2.8% at 100 mg·L^−1^ PBZ (*p* < 0.05), but decreased to 36.8 ± 4.5% at 200 mg·L^−1^ PBZ (*p* < 0.05). PBZ-treated cuttings exhibited significantly more callus tissue at the base, which correlated with reduced root quality (Figure 1B,C). High-dose PBZ (200 mg·L^−1^) caused severe callus overgrowth, inhibiting root elongation (Figure 1C).

### 2.2. GA_4_ Dynamics During Rooting

**Figure 2:**
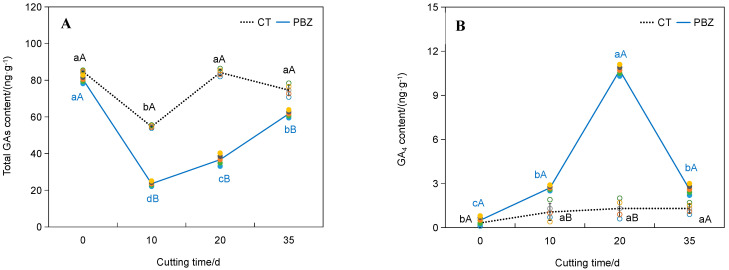

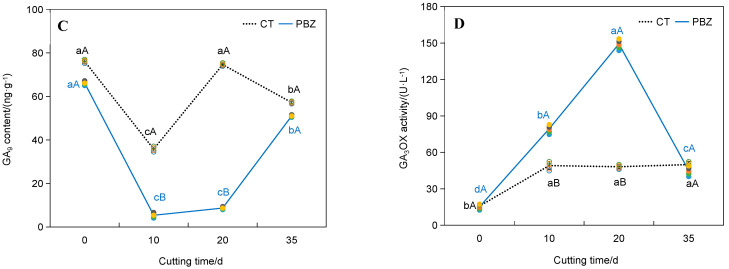
GA dynamics and critical enzyme activity during rooting in *Pinus massoniana* cuttings under PBZ treatment. Raw data for active GAs and inactive precursors detected in this work were recorded in Table S1. The circles represent individual replicates for each treatment. Hollow circles: CT, 200 mg·L^−1^ NAA; Solid circles: PBZ, 200 mg·L^−1^ NAA + 100 mg·L^−1^ PBZ. Line plots depict the mean values. Dashed line: CT; Continuous line: PBZ. (**A**) the total content of 16 GAs; (**B**) GA_4_ content; (**C**) GA_9_ content; (**D**) GA_3_OX activity. Lowercase letters (a, b, c, d) denote significant differences among cutting time; uppercase letters (A, B) indicate differences between treatments at the same time point (Tukey’s HSD, *p* < 0.05; *t*-test, *p* < 0.05).

**Figure 3:**
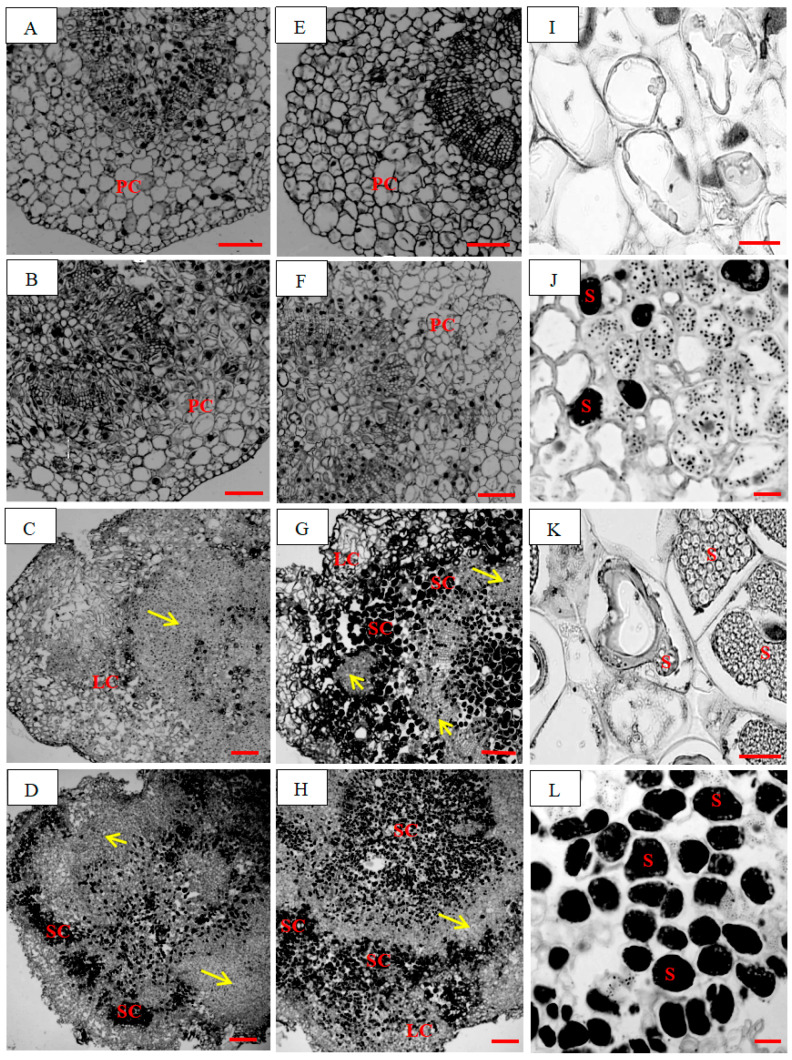
Anatomy of *Pinus massoniana* cuttings during rooting under GA_4_ treatment. (**A**–**H**) anatomical structure of adventitious roots during the cutting of 35 days ((**A**,**E**) cutting 0 d; (**B**,**F**) cutting 10 d; (**C**,**G**) cutting 20 d; (**D**,**H**) cutting 35 d). The arrow shows the induced root primordium. (**I**–**L**) suberin micrograph after 35-day cutting. (**A**–**D**,**I**,**J**) CT (200 mg·L^−1^ NAA); (**E**–**H**,**K**,**L**) GA_4_ (200 mg·L^−1^ NAA + 100 mg·L^−1^ GA_4_). PC, parenchyma cells. S, suberin. LC, lignified cells. SC, Suberized cells. Representative images were from 5 independent sections, and suberin deposition was quantified as area fraction (%) using ImageJ software. Scale bar: (**A**–**H**) 200 μm; (**I**–**L**) 20 μm.

**Figure 4:**
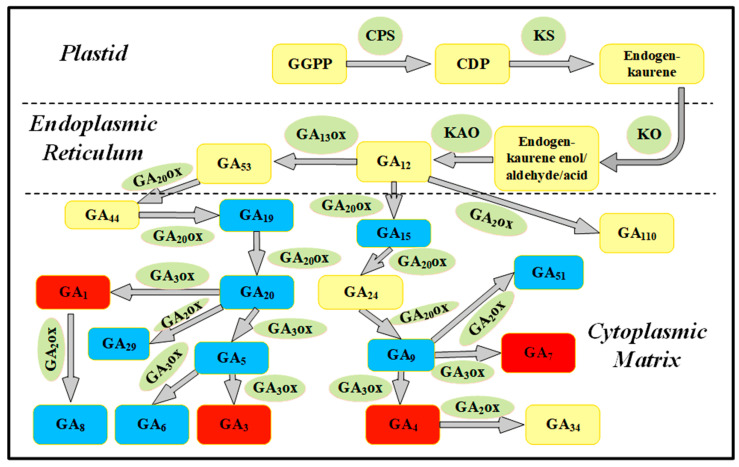
The gibberellin biosynthesis metabolic pathway in *Pinus massoniana* cuttings. Green boxes represent enzymes, yellow boxes represent undetected gibberellins (GAs), and the detected GAs are highlighted with blue and red boxes in this work. Blue boxes represent inactive precursor GAs, and red boxes represent active GAs. This study focused on GA_4_ because its content was significantly elevated under paclobutrazol treatment (200 mg·L^−1^ NAA + 100 mg·L^−1^ PBZ) at 20 days after cutting—a stage associated with the arrest of root development and onset of suberization—while other active GAs (GA_1_, GA_3_, GA_7_) levels remained unchanged compared to the control treatment (200 mg·L^−1^ NAA) (Table S1). This implicates GA_4_ as a potential key regulator of the rooting difficulty phenotype. The simplified pathway is based on well-established routes in plants, and arrows indicate multiple enzymatic steps.

Although the total content of 16 endogenous GAs decreased by 2.3-fold after 10–20 day PBZ treatment (Figure 2A, *p* < 0.05), endogenous GA_4_ levels exhibited stage-specific upregulation (Figure 2B). At the early stage (0–10 days), GA_4_ content did not significantly increase under PBZ treatment (*p* > 0.05), coinciding with slightly enhanced GA_3_OX activity by 62.3% (*p* > 0.05) and decreased GA_9_ content by 7.6-fold (*p* < 0.05). At the late stage (20–35 days), GA_4_ accumulation was increased by 723.1% under PBZ treatment (*p* < 0.05), paralleling remarkably upregulated GA_3_OX activity by 210.9% (*p* < 0.05), downregulated GA_9_ content by 8.6-fold (*p* < 0.05) (Figure 2B–D), and suberization intensification (Figure 3G,H). This coordinated metabolic shift suggests that PBZ-mediated GA_9_ depletion may redirect metabolic flux toward GA_4_ synthesis via GA_3_OX upregulation (Figure 4). This aberrant elevation of GA_4_ levels during the late PBZ treatment phase may reflect metabolic reprogramming rather than simple biosynthetic induction, as evidenced by the GA_3_OX activity-mediated regulatory mechanism.

### 2.3. GA_4_ Inhibits Root Development

**Figure 5:**
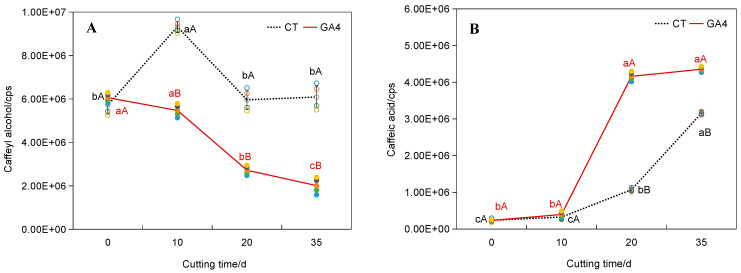

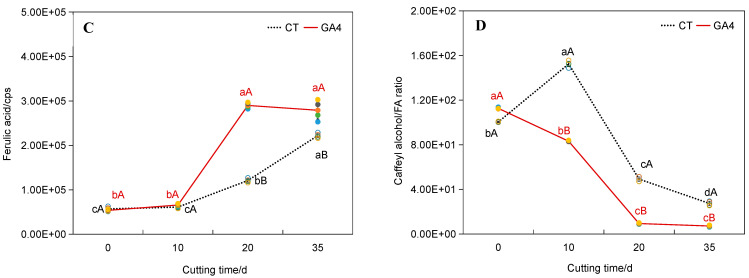
Lignin precursors profiling in *Pinus massoniana* cuttings under GA_4_ treatment. Raw data for lignin precursors identified in this work were recorded in Table S1. The circles represent individual replicates for each treatment. Hollow circles: CT, 200 mg·L^−1^ NAA; Solid circles: GA_4_, 200 mg·L^−1^ NAA + 100 mg·L^−1^ GA_4_. Line plots depict the mean values. Dashed line: CT; Continuous line: GA_4_. The ordinate unit cps in the figure is the abbreviation of counts per second, indicating the number of small lignin molecules detected per second in the sample. (**A**) caffeyl alcohol; (**B**) caffeic acid; (**C**) ferulic acid. (**D**) caffeoyl alcohol/FA ratio, which was decreased by 45.3–81.0% under GA_4_ treatment (*p* < 0.05), supporting the substrate competition hypothesis. Lowercase letters (a, b, c, d) denote significant differences among cutting time; uppercase letters (A, B) indicate differences between treatments at the same time point (Tukey’s HSD, *p* < 0.05; *t*-test, *p* < 0.05).

Exogenous GA_4_ application (100–200 mg·L^−1^) negatively impacted rooting (Table 2). Rooting percentage decreased by 66.4–69.2% compared to control (*p* < 0.05). Suberin deposition was significantly increased by 3.2-fold in GA_4_-treated cuttings (*p* < 0.05; Figure 3K,L), visualized by oil lens and toluidine blue staining, and quantified via ImageJ 2.0.0-rc-54 (area fraction, %). Metabolic profiling demonstrated that 100 mg·L^−1^ GA_4_ treatment reduced caffeoyl alcohol content by 54.4% (*p* < 0.05, Figure 5A) at root development stage (20 days), while concomitantly increasing caffeic acid and FA levels by 2.4–3.9-fold (*p* < 0.05, Figure 5B,C), resulting in the decrease in alcohol/FA ratio by 81.0% (*p* < 0.05, Figure 5D). These metabolic alterations strongly indicate that GA_4_ induces a competition between lignin and suberin biosynthesis, as evidenced by the inverse relationship between lignin precursors (caffeoyl alcohol) and suberin components (FA/caffeic acid).

### 2.4. FA-Mediated ROS Homeostasis and Its Effects on Rooting

**Figure 6:**
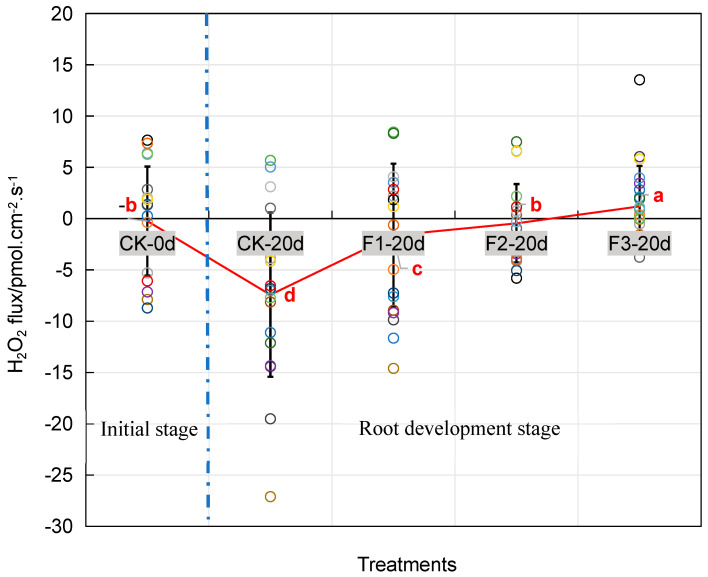
The net flux of H_2_O_2_ in *Pinus massoniana* cuttings under FA treatment. Ten minutes of data were recorded (Table S1). The hollow circles represent the H_2_O_2_ flux at each individual measurement time point. The line plot depicts the mean value of all H_2_O_2_ flux measurements over time. Sampled cuttings were collected from cutting 0/20 days and four FA treatments: CT-0 d, cutting 0 day and 200 mg·L^−1^ NAA treatment; CT-20 d, cutting 20 day and 200 mg·L^−1^ NAA treatment; F1-20 d, cutting 20 day and 200 mg·L^−1^ NAA + 200 mg·L^−1^ FA; F2-20 d, cutting 20 day and 200 mg·L^−1^ NAA + 600 mg·L^−1^ FA (F2); F3-20 d, cutting 20 day and 200 mg·L^−1^ NAA + 1000 mg·L^−1^ FA. Negative values (−) represent net influx (uptake) of H_2_O_2_ into the tissue, while positive values (+) represent net efflux (release). Different lowercase letters (a, b, c, d) indicate significant differences among four FA treatments (Tukey’s HSD, *p* < 0.05).

**Figure 7:**
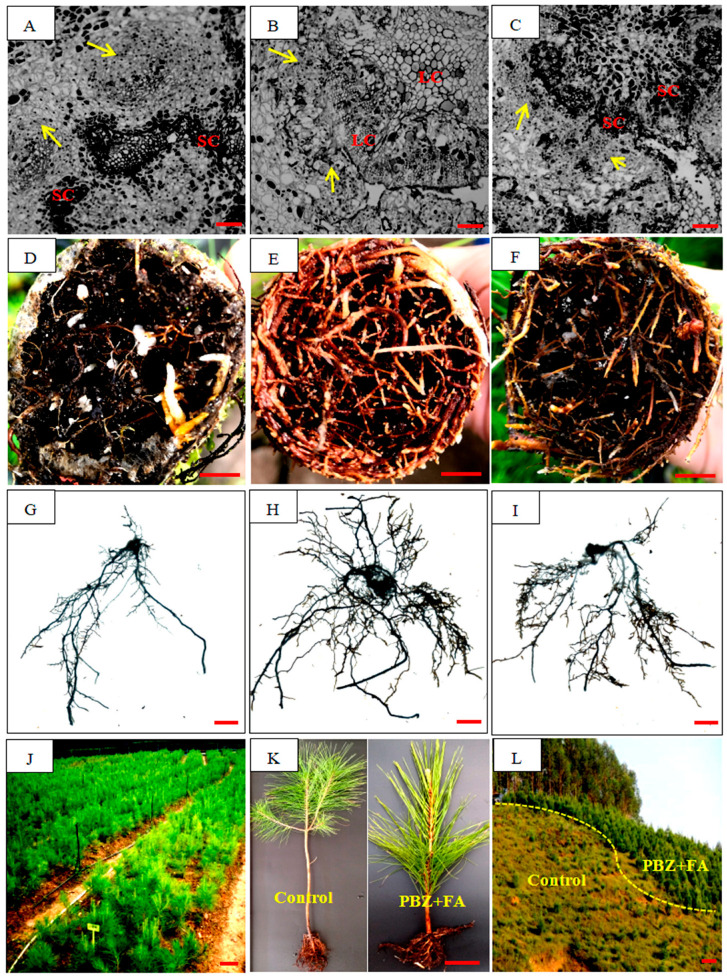
Effects of FA or FA + PBZ on rooting of *Pinus massoniana* cuttings. (**A**–**C**) anatomy of cuttings at root formation stage (cutting 35 d) under FA treatment, and the arrow shows the induced root primordium. (**A**) CT (200 mg·L^−1^ NAA), (**B**) F1 (200 mg·L^−1^ NAA + 200 mg·L^−1^ FA), (**C**) F2 (200 mg·L^−1^ NAA + 600 mg·L^−1^ FA); (**D**–**I**): root morphology of cuttings after 2-months cutting under FA treatment, (**D**,**G**) CT (200 mg·L^−1^ NAA), (**E**,**H**) F1 (200 mg·L^−1^ NAA + 200 mg·L^−1^ FA), (**F**,**I**) F2 (200 mg·L^−1^ NAA + 600 mg·L^−1^ FA); (**J**–**L**): Large-scale production and application of cutting seedlings through the combined use of 200 mg·L^−1^ NAA + 100 mg·L^−1^ PBZ + 200 mg·L^−1^ FA, (**J**) scion orchard; (**K**) cutting seedlings after 5 months of cutting; (**L**) 2-year-old clonal forest. LC, lignified cells. SC, Suberized cells. Scale bar: (**A**–**C**) 200 μm; (**D**–**I**) 1 cm; (**J**) 15 cm; (**K**) 5 cm; (**L**) 2 m.

In contrast with the initial stage of cutting, a sharp increase in H_2_O_2_ was investigated at the root emergence zone after 20-day cutting (root development stage) (Figure 6). Hence, effects of FA on H_2_O_2_ dynamics at root development stage were explored. As was shown in Figure 6, FA treatments (200–1000 mg·L^−1^) dose-dependently attenuated the net H_2_O_2_ influx (i.e., the flux values became less negative) by 77.9–116.1% compared to control (*p* < 0.05). To FA-dose response, 200 mg·L^−1^ FA optimized H_2_O_2_ balance, improving rooting percentage by 15.9% (*p* < 0.05), while 1000 mg·L^−1^ FA led to the complete H_2_O_2_ influx suppression and 21.6% root inhibition (*p* < 0.05) (Figure 6, Table 3). Non-invasive Micro-test Technology (NMT) imaging revealed FA-mediated ROS compartmentalization in rhizome tissues. For the effects of FA on rooting, dose-dependent effects of FA were observed. Low-dose FA (200 mg·L^−1^) alleviated root suberization and improved root quality, while high-dose FA (600 mg·L^−1^) exacerbated suberin accumulation, leading to poor root morphology (Figure 7A–I).

### 2.5. Synergistic Effects of PBZ and FA Combination

The combined application of PBZ and FA (PBZ + FA) significantly enhanced rooting efficiency compared to the control (CT, NAA alone) (Table 4). Specifically, rooting percentage increased by 34.4%, and rooting time was reduced by 18.4% (both *p* < 0.05). After two years, field survival rates reached 95.8% for PBZ + FA treatment, markedly surpassing the 68.5% in the control (NAA, *p* < 0.05). 95.8% field survival (Table 4) underscores protocol efficacy, demonstrating unprecedented clonal establishment success. However, this combination decreased root number to 5.8 ± 1.4 roots/plant, which was significantly lower than the FA group (8.2 ± 2.6 roots/plant, *p* < 0.05)-with root number inversely correlating with suberin area fraction (r = −0.82, *p* < 0.05; Figure 3L), further supporting the tradeoff between PBZ and FA. Mechanistically, PBZ + FA synergistically suppressed GA_4_-induced suberization while maintaining redox homeostasis. This dual action facilitated efficient large-scale production and field application of cuttings (Figure 7J–L).

## 3. Discussion

Our study uncovers a previously unrecognized regulatory axis governing GA-mediated adventitious rooting recalcitrance in *P. massoniana*, integrating hormonal crosstalk, redox homeostasis, and cell wall remodeling. The discovery of a GA_4_-FA-ROS-suberin signaling network not only provides evidence for a novel mechanism in GA function in conifers but also provides a mechanistic foundation for optimizing clonal propagation in recalcitrant forest trees.

Contrary to the established role of GAs as general growth promoters, our findings reveal a stage-specific and concentration-dependent dichotomy in GA action during adventitious rooting. While PBZ-mediated GA depletion accelerates rooting kinetics (Figure 1), the late-stage accumulation of GA_4_ under PBZ treatment (Figure 2B) paradoxically arrests root development. This biphasic effect, linked to dynamic equilibrium in the GA biosynthetic pathway, is mechanistically underpinned by GA_3_OX-mediated metabolic rerouting: PBZ-induced depletion of GA_9_ (a GA_4_ precursor) diverts flux toward GA_4_ synthesis via upregulated GA_3_OX activity (Figure 4). Such metabolic plasticity highlights an overlooked layer of GA regulation in gymnosperms, where substrate availability and enzyme kinetics synergize to shape hormone action. Notably, the 210.9% activation of GA_3_OX under PBZ parallels the 723.1% surge in GA_4_ (Figure 2B). The parallel increase may indicate a feedforward loop between GA_3_OX and GA_4_. This contrasts with angiosperms, where GA_3_OX typically acts as a rate-limiting enzyme [3,8]. Conifer-specific GA metabolic plasticity may explain preferential GA_4_ accumulation. The differential regulation in conifers may reflect an evolutionary adaptation to their unique developmental programs, such as prolonged juvenile phases and stress-induced dormancy [9,10], though comparative studies are needed. Future studies should explore whether this metabolic rewiring is conserved across Pinaceae species.

**Figure 8:**
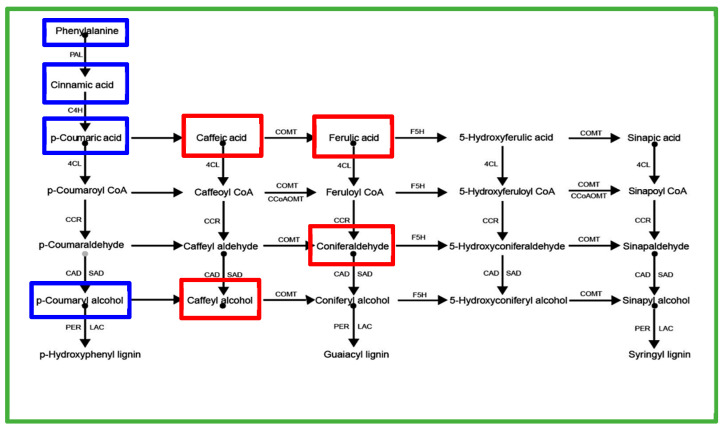
Proposed G-type lignin biosynthesis pathway in *Pinus massoniana* rhizomes. Lignin precursors identified in this work are highlighted. Blue frames: precursors whose content did not differ significantly between CT (200 mg·L^−1^ NAA) and GA_4_ (200 mg·L^−1^ NAA + 100 mg·L^−1^ GA_4_) treatments at 20 days after cutting. Red frames: precursors whose content differed significantly between CT and GA_4_ treatments at 20 days after cutting.

The identification of FA as a critical mediator of GA_4_-induced suberization represents a paradigm shift in our understanding of conifer root development. While lignification is traditionally viewed as the primary barrier to rooting in woody plants [11], our data reveal a previously unappreciated role for suberin deposition in GA_4_-treated cuttings (Figure 3K,L). We provide the first evidence that the GA_4_-FA axis regulates adventitious root formation in conifers through ROS-mediated switching between suberin and lignin metabolism, offering novel targets to overcome rooting barriers in woody plants. At root development stage, the 54.4% reduction in caffeoyl alcohol (Figure 5B)—a G-type lignin precursor (Figure 8)—and the concurrent 2.4–3.9-fold accumulation of FA and caffeic acid (Figure 5C,D) suggest a substrate competition mechanism between lignin and suberin biosynthesis pathways, given that FA and caffeic acid are essential components of suberin [12,13]. This finding resonates with emerging evidence that monolignol-derived phenolics can be shunted into suberin synthesis under stress conditions [14]. The spatial correlation between GA_4_ accumulation and suberin deposition in rhizome tissues (Figure 3G,H) implies a direct regulatory link, potentially mediated by transcriptional reprogramming of genes involved in phenylpropanoid metabolism (e.g., PAL, C4H, CCR) [15,16]. While phenylpropanoid pathway enzymes are mentioned (PAL, C4H, CCR), no actual gene expression data or profiling was performed in the study. Future studies should verify these metabolic routes through RNA-seq or proteomic analysis.

Plant regulation of suberization serves as an adaptive strategy to environmental stresses [12,17]. ROS can act as signaling molecules that participate in suberization regulation via hormonal and metabolic pathways (e.g., ABA) [18]. However, excessive ROS accumulation may induce cellular damage, triggering suberization as a response to oxidative stress. Therefore, ROS is considered one of the direct inducers of suberization [19]. FA modulates ROS levels through multiple mechanisms (direct scavenging of ROS, activation of antioxidant pathways, and regulation of gene expression) and serves as a critical metabolic intermediate in suberization by participating in structural formation during this process [12,13,20]. FA reduced net H_2_O_2_ influx, consistent with ROS-scavenging activity. In this study, the dual role of FA in modulating H_2_O_2_ flux (Figure 6) and suberin deposition (Figure 7) uncovers a redox-based signaling hub governing root development. At low doses (200 mg·L^−1^), FA acts as a ROS scavenger, reducing net H_2_O_2_ influx by 93.7% (Figure 6) and stabilizing redox homeostasis to promote root induction. Conversely, high-dose FA (1000 mg·L^−1^) induces ROS depletion, suppressing H_2_O_2_ influx entirely and disrupting redox-sensitive signaling pathways required for cell division. This biphasic effect aligns with the hormetic dose–response model, where ROS functions as a double-edged sword in plant development [4]. Critically, the FA-mediated ROS compartmentalization (Figure 7B) provides a mechanistic explanation for the observed tradeoff between rooting quantity and quality. Low ROS levels favor cell expansion and differentiation, whereas excessive ROS scavenging impairs mitotic activity [21]. This paradigm resonates with previous studies demonstrating that ROS gradients dictate root meristem size [22].

PBZ’s auxin dependency confirms hormonal synergism is non-redundant, contrasting with angiosperm models [2]. PBZ alone did not induce rooting (Table 1), confirming that its inhibition of GA needs to be coupled with auxin signals to promote root primordium differentiation. In the presence of auxin NAA, the synergistic interaction between PBZ and FA (Table 4, Figure 7J–L) demonstrates a novel combinatorial strategy to overcome recalcitrant rooting in conifers by simultaneously targeting GA_4_-mediated suberization and ROS dynamics. While PBZ effectively suppressed GA_3_ synthesis to enhance initial rooting rates (as previously validated [6]), our findings revealed an unexpected GA_4_ upregulation during root development, which exacerbated suberization through elevated FA and caffeic acid levels, key components of suberin and antioxidants. This mechanistic conflict necessitated the integration of FA, which exhibited dual-phase regulation: at moderate concentrations, FA buffered ROS spikes during root primordium development (Figure 6), while its controlled degradation prevented excessive ROS quenching that could paradoxically promote suberogenesis. The 34.4% rooting improvement and 95.8% two-year survival (Figure 7L) underscore the optimized balance achieved through this dual-targeting approach. However, the 31.7% root number reduction compared to FA-monotherapy (Table 4) may stem from GA_4_-induced suberization or FA toxicity thresholds.

The discovery of GA_4_-suberin crosstalk in *P. massoniana* offers insights into the evolutionary pressures shaping conifer reproductive strategies. The propensity for GA_4_ accumulation during late rooting stages may reflect an adaptive mechanism to prevent precocious rooting in fire-prone ecosystems, where delayed rooting confers drought tolerance [23]. Conversely, FA-mediated suberization could serve as a wound-response mechanism, sealing damaged tissues to prevent pathogen ingress [24]. While adaptive mechanisms are hypothesized, genotype-specific validation beyond GLM-3 is essential. From an applied perspective, our findings reconcile conflicting reports on PBZ’s efficacy in conifer propagation. While PBZ has been dismissed as ineffective in some studies [25], our results demonstrate that its utility hinges on precise dose-timing and combinatorial treatments. This aligns with the emerging concept of “hormonal choreography” in plant tissue culture [26], where sequential hormone applications are required to navigate developmental checkpoints.

Our study paves the way for precision propagation protocols in commercial forestry. The PBZ + FA combination achieves a 2.3-fold increase in field survival (Figure 7L), addressing the critical barrier to clonal deployment of superior genotypes. Scaling this technology will require addressing two key challenges: (1) standardizing GA_3_ox activity assays for routine nursery diagnostics and (2) developing nanoformulations to deliver PBZ and FA in spatiotemporally controlled manners. Moreover, the GA_4_-suberin axis presents novel targets for genetic engineering. RNAi-mediated silencing of GA_3_OX or overexpression of GA_2_OX (an active GA catabolism enzyme, Figure 4) could enhance rooting efficiency, while CRISPR-based repression of suberin biosynthesis genes (e.g., GPAT, LACS) [27,28] may bypass the need for exogenous regulators. Such genome-editing strategies are particularly relevant given the slow progress in conifer transformation pipelines. In this study, the possibility of cross-regulation of cytokinins by PBZ was not completely ruled out. Subsequently, metabolomics and gene editing techniques will be combined to deeply analyze the mechanism of multi-hormone synergy.

## 4. Materials and Methods

### 4.1. Plant Materials

Mother trees of *P. massoniana* were selected from a 15-year-old plantation in Paiyangshan Forest Farm (Chongzuo, Guangxi, China; 22°25′35″ N, 109°22′19″ E, elevation 680 m). Trees exhibited superior growth (DBH: 18–22 cm, height: 13–18 m) and no signs of pests/diseases. Micrografting was performed following the protocol of Wang et al. [29] with modifications. Terminal shoots were derived from 5-month-old grafted seedlings of the clone GLM-3 (selected for its consistent root response in prior studies and commercial relevance in subtropical China) (Guangxi Forestry Research Institute, Nanning, China). Experiments were repeated in October 2019–2021 with identical protocols. Explants were surface-sterilized with 75% ethanol for 30 s followed by rooting agent treatment for 4 h, then transferred into rooting substrate, a 1:1:1 (*v*/*v*/*v*) mixture (Guangxi Forestry Research Institute, Nanning, China) of peat soil, perlite, and coconut bran, which was autoclaved at 121 °C for 2 h prior to use. Substrate moisture content was maintained at 60–70% throughout the experiment.

### 4.2. Rooting Agent Treatment

Terminal shoots (15 cm length) were treated with various combinations of NAA, PBZ, GA_4_, and FA. Concentrations of NAA, PBZ, GA_4_, and FA were selected based on preliminary dose–response trials and prior studies in conifers [6,8]. Detailed concentration gradients and sampling time points for all treatment groups were summarized comprehensively in Supplementary Table S1, ensuring full transparency of experimental parameters.

#### 4.2.1. PBZ Treatment

Cuttings from different grafted seedlings were randomly assigned to seven treatment groups in a randomized block design across greenhouse sections to minimize pseudo-replication. The groups were as follows: the control treatment (CT), consisted of a basal application of 200 mg·L^−1^ NAA; three NAA + PBZ treatment groups, treated with 200 mg·L^−1^ NAA combined with 50, 100, or 200 mg·L^−1^ PBZ (Sigma-Aldrich, St. Louis, MO, USA, ≥98% purity); and three PBZ treatment groups, 50, 100, or 200 mg·L^−1^ PBZ (no NAA). Each treatment group consisted of five biological replicates, with each replicate containing 70 cuttings. PBZ and/or NAA solutions were prepared in deionized water and applied via basal soaking for 4 h. Cuttings were incubated in a greenhouse (108°22′ E, 22°55′ N) under natural light (72–90 μmol·m^−2^·s^−1^), ambient temperature (25 ± 2 °C), natural photoperiod (~12 h light/dark cycle), CO_2_ levels ~400 ppm, and misted every 2 h to maintain humidity > 90%. PBZ effects were assessed at 0 (initial stage), 10 (root induction stage), 20 (root development stage), and 35 (root formation stage) days post-treatment.

#### 4.2.2. GA_4_ Treatment

Cuttings (five biological replicates, 70 cuttings per replicate) were exposed to four GA_4_ treatments: 200 mg·L^−1^ NAA (control, CT); 200 mg·L^−1^ NAA + 50/100/200 mg·L^−1^ GA_4_ (Sigma-Aldrich, St. Louis, MO, USA, ≥98% purity). Solutions were prepared in deionized water and used following the same method as PBZ treatment.

#### 4.2.3. FA Treatment

Cuttings (five biological replicates, 70 cuttings per replicate) were treated with four FA concentrations, control (CT): 200 mg·L^−1^ NAA; F1–F3: 200 mg·L^−1^ NAA supplemented with 200, 600, or 1000 mg·L^−1^ FA (Sigma-Aldrich, St. Louis, MO, USA, ≥99% purity). Solutions were prepared in deionized water and applied as the methodology of PBZ treatment.

#### 4.2.4. PBZ + FA Treatment

To optimize the rooting agents and evaluate their application, cultivation effects of cutting seedlings at large scales were compared among four treatments of rooting agents, CT: 200 mg·L^−1^ NAA (control); PBZ: 200 mg·L^−1^ NAA + 100 mg·L^−1^ PBZ; FA: 200 mg·L^−1^ NAA + 200 mg·L^−1^ FA; PBZ + FA: 200 mg·L^−1^ NAA + 100 mg·L^−1^ PBZ + 200 mg·L^−1^ FA. Treatments were applied following the same protocols as individual PBZ/FA treatments.

### 4.3. Rooting Performance Assessment

Rooting parameters of cuttings from PBZ/GA_4_/FA/PBZ + FA treatments were evaluated at multiple time points. Rooting time was recorded as the number of days from treatment initiation to visible root emergence (>2 mm). Rooting percentage was calculated as the ratio of rooted cuttings to total cuttings at 2 months post-treatment. Root number was counted as the number of roots > 2 cm per plant at 2 months. Root morphology traits: number (>2 cm), length, and callus index were documented. Root quality was assessed by two parameters: callus-to-root ratio (callus area ÷ root number), and root elongation capacity (percentage of roots > 2 cm in length). Nursery survival percentage was determined at 5 months post-treatment as the ratio of surviving plants to rooted plants at 2 months. Field survival percentage was assessed after 2 years of field cultivation based on plant vigor, crown integrity, and disease resistance.

### 4.4. GA Quantification (LC-MS/MS)

Basal stem segments (1 cm length, 0.5 g fresh weight per replicate, n = 5) were homogenized for hormone quantification as they contain the root primordia initiation sites [11]; apical/root zones showed negligible GA fluctuations in pilot assays. The segments were ground in liquid nitrogen, extracted with 80% methanol containing 0.1% formic acid (*v*/*v*), and sonicated (40 kHz, 30 min), and then extracts were purified using Oasis HLB SPE columns (Waters, Milford, MA, USA) and dried under vacuum. Data normalized to fresh weight (ng·g^−1^ FW).

LC-MS/MS Conditions: Column, ACQUITY HSS T3 (1.8 μm, 100 mm × 2.1 mm); Mobile Phase, A (0.04% formic acid in water)/B (0.04% formic acid in acetonitrile); Gradient, 0 min (95% A), 10 min (5% A), 11 min (95% A), 14 min (stop); Flow Rate, 0.35 mL·min^−1^, Column Temp: 40 °C, Injection Vol: 10 μL; ESI-MS: Positive ion mode, Curtain Gas 35 psi, Ion Spray Voltage 5500 V.

Quantification: Quantification used external standards (Olchemim, Olomouc, Czech Republic) and isotope-labeled internal standards (16 kinds of GAs). Method validation included linearity (R^2^ > 0.99), LOD (0.1–1 pg), and intra-day precision (<15%).

### 4.5. GA_3_OX Activity Assay

GA_3_OX activity was determined using a double-antibody sandwich ELISA method. Basal stem segments were homogenized in 1.8 mL ice-cold PBS (pH 7.4). The homogenate was centrifuged at 5000× *g* for 15 min at 4 °C. The supernatant was collected for analysis. Pre-coated ELISA plates were equilibrated at room temperature (15–30 °C) for 15 min. Serial dilutions of standards (1–16 μL) were prepared using provided diluents. Add 10 μL standards/samples to wells, and incubate with 40 μL sample diluent and 50 μL enzyme conjugate at 37 °C for 30 min. Wash wells 5× with buffer after primary and secondary incubations, add 50 μL chromogenic reagents A and B sequentially, and then incubate at 37 °C in the dark for 10 min. Terminate reaction with stop solution and measure OD450 nm within 15 min. Standard curves were generated using Excel to calculate GA_3_OX concentrations in samples.

### 4.6. Anatomical Observation

Rhizome sections (1 cm below the base) were fixed in FAA (formalin:acetic acid:ethanol = 5:5:90), dehydrated in graded ethanol (30–100%), embedded in paraffin, and sectioned (8 μm). Sections were stained with safranin O/fast green and observed under a Eclipse Ti2 microscope (Nikon, Tokyo, Japan). Suberin deposition was visualized via toluidine blue staining (pH 4.5) and quantified using ImageJ software (area fraction of stained tissue, %).

### 4.7. Lignin Precursors Profiling

Lignin precursors (14 compounds) were quantified using the protocol from Wang et al. [6] with isotopically labeled internal standards (e.g., ^13^C-caffeic acid and ^13^C-ferulic acid; Cambridge Isotope Laboratories, Tewksbury, MA, USA) to correct for matrix effects, with 85–110% recovery rates. Methanol:water:formic acid (15:4:1, *v*/*v*/*v*) was used for ultrasonic-assisted extraction. LC-MS/MS parameters were modified from the GA method (same column, gradient adjusted for polar compounds).

### 4.8. H_2_O_2_ Flux Analysis (NMT)

Hydrogen peroxide fluxes were measured using an NMT system (Xuyue SciTech, Beijing, China) as described in our previous study [30] at 20 days after FA post-treatment. Negative values (−) represent net influx (uptake) of H_2_O_2_ into the tissue, while positive values (+) represent net efflux (release). For calibration, standard curves (0.1–10 μM H_2_O_2_) were established daily. Sampling frequency was 0.1 Hz, and electrode tip diameter was <1 μm.

### 4.9. Statistical Analysis

All quantitative data are presented as mean ± standard deviation (SD) from [n] biological replicates. Statistical comparisons between two groups were performed using an unpaired *t*-test. For comparisons among three or more groups, one-way analysis of variance (ANOVA) was applied, followed by Tukey’s HSD post hoc test for multiple comparisons if the ANOVA result was significant. Prior to ANOVA, the validity of the key parametric assumptions was rigorously checked. The normality of the residuals for each dataset was confirmed using the Shapiro–Wilk test (all *p* > 0.05), and the homogeneity of variances across groups was verified using Levene’s test (all *p* > 0.05). In full alignment with robust statistical practice and to guard against any potential concerns regarding sample size, all significant findings were further validated using the non-parametric Kruskal–Wallis test followed by Dunn’s test with appropriate adjustment for multiple comparisons. The outcomes of both parametric and non-parametric analyses were entirely consistent in terms of significant effects across all tested hypotheses. Differences were considered statistically significant at *p* < 0.05. All statistical analyses were conducted using SPSS v26 (IBM, New York, NY, USA).

## 5. Conclusions

**Figure 9:**
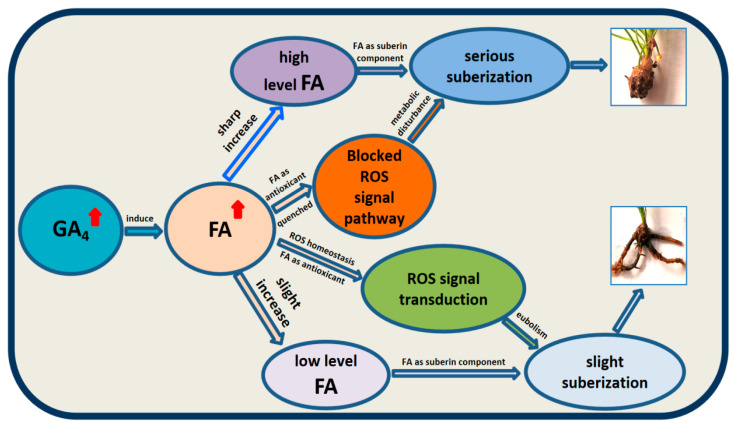
The GA_4_-FA-ROS-suberin regulatory axis. GA_4_ upregulation diverts phenylpropanoid flux toward suberin via FA accumulation. FA optimizes ROS homeostasis at low doses (200 mg·L^−1^) but inhibits mitosis at high doses (1000 mg·L^−1^). The red arrow denotes a substantial mass gain.

This study elucidates a GA_4_-mediated regulatory network underlying adventitious rooting recalcitrance in *P. massoniana*. PBZ optimized rooting kinetics, while it redirected gibberellin flux through GA_3_OX upregulation, resulting in GA_4_ accumulation. Exogenous GA_4_ inhibited root development by inducing suberization via FA-mediated redox imbalance. Combining PBZ with FA synergistically improved rooting efficiency (34.4% increase) and field survival (95.8% after 2 years) by suppressing GA_4_-induced suberization and stabilizing ROS homeostasis. These findings firstly reveal a previously unrecognized GA_4_-FA-ROS-suberin signaling axis (Figure 9) in gymnosperms, providing a new target for breaking through the rooting obstacles of woody plants. The study develops a scalable protocol for clonal propagation of recalcitrant conifers and highlights FA as a dual-function regulator integrating redox signaling and cell wall remodeling. Future research should focus on manipulation of GA_3_OX and suberin biosynthesis genes to further enhance propagation efficiency.

## Figures and Tables

**Table 1:** Rooting performance of *Pinus massoniana* cuttings under PBZ treatment.

NAA Concentration /mg·L^−1^	PBZ Concentration /mg·L^−1^	Rooting Percentage /%	Rooting Time /d	Root Number Per Plant />2 cm	Nursery Survival Percentage /%
200	0	58.6 ± 3.3 c	45.2 ± 1.5 a	1.9 ± 0.4 a	52.3 ± 3.6 a
200	50	67.5 ± 1.8 b	40.2 ± 2.2 b	2.2 ± 0.3 a	53.8 ± 3.9 a
200	100	79.5 ± 2.8 a	30.5 ± 2.3 c	2.0 ± 0.5 a	56.3 ± 4.1 a
200	200	36.8 ± 4.5 d	31.5 ± 3.7 c	1.1 ± 0.7 a	33.6 ± 5.7 b
0	50~200	0	-	-	-

Note: different lowercase letters in the table indicate significant differences between rooting agent treatment. The data presentation was in the form of mean ± SD (n = 5). Data analyzed by ANOVA with Tukey’s HSD; homogeneity of variance confirmed via Levene’s test. PBZ-alone vs. PBZ + NAA: rooting percentage differed by 79.5% (*p* < 0.05). PBZ treatment alone (without NAA) had no rooting effect, indicating that its synergistic effect with auxin was a necessary condition.

**Table 2:** Rooting performance of *Pinus massoniana* cuttings under GA_4_ treatment.

NAA Concentration /mg·L^−1^	GA_4_ Concentration /mg·L^−1^	Rooting Percentage /%	Rooting Time /d	Root Number Per Plant />2 cm	Nursery Survival Percentage /%
200	0	53.2 ± 3.4 a	45.9 ± 2.7 a	2.2 ± 0.4 a	56.4 ± 3.8 a
200	50	50.4 ± 2.5 a	45.1 ± 2.5 a	2.3 ± 0.4 a	58.8 ± 3.7 a
200	100	16.4 ± 2.4 b	47.2 ± 1.7 a	1.1 ± 0.2 b	22.3 ± 2.1 b
200	200	17.9 ± 3.8 b	46.5 ± 2.8 a	1.2 ± 0.2 b	20.9 ± 1.8 b

Note: different lowercase letters in the table indicate significant differences between rooting agent treatments. The data presentation was in the form of mean ± SD (n = 5). Data analyzed by ANOVA with Tukey’s HSD; homogeneity of variance confirmed via Levene’s test.

**Table 3:** Rooting performance of *Pinus massoniana* cuttings under FA treatment.

Treatment	NAA Concentration /mg·L^−1^	FA Concentration /mg·L^−1^	Rooting Percentage /%	Rooting Time /d	Root Number Per Plant />2 cm	Nursery Survival Percentage /%
CT	200	0	54.2 ± 3.1 b	46.3 ± 2.4 b	2.4 ± 0.7 c	51.4 ± 4.4 c
F1	200	200	62.8 ± 2.5 a	39.3 ± 3.5 c	8.9 ± 1.5 a	86.4 ± 3.8 a
F2	200	600	44.8 ± 5.5 c	50.5 ± 2.2 a	4.1 ± 0.2 b	62.5 ± 2.4 b
F3	200	1000	42.5 ± 3.8 c	52.8 ± 4.3 a	1.2 ± 0.4 d	37.9 ± 3.2 d

Note: different lowercase letters in the table indicate significant differences between rooting agent treatments. FA, ferulic acid. The data presentation was in the form of mean ± SD (n = 5). Data analyzed by ANOVA with Tukey’s HSD; homogeneity of variance confirmed via Levene’s test.

**Table 4:** Rooting performance of *Pinus massoniana* cuttings under PBZ + FA treatment.

Treatment	NAA Concentration /mg·L^−1^	PBZ Concentration /mg·L^−1^	FA Concentration /mg·L^−1^	Rooting Percentage /%	Rooting Time /d	Root Number Per Plant />2 cm	Nursery Survival Percentage /%	Field Survival Percentage /%
CT	200	0	0	52.5 ± 3.6 d	45.0 ± 1.7 a	2.0 ± 0.5 c	55.2 ± 3.7 c	68.5 ± 4.2 b
PBZ	200	100	0	78.8 ± 2.9 a	31.6 ± 3.8 c	1.9 ± 0.8 c	54.8 ± 3.3 c	70.2 ± 5.6 b
FA	200	0	200	61.5 ± 2.1 c	38.2 ± 2.9 b	8.2 ± 2.6 a	88.9 ± 2.6 a	97.3 ± 2.1 a
PBZ + FA	200	100	200	70.6 ± 3.6 b	36.7 ± 2.8 b	5.8 ± 1.4 b	80.2 ± 2.5 b	95.8 ± 2.9 a

Note: different lowercase letters in the table indicate significant differences between rooting agent treatments. The data presentation was in the form of mean ± SD (n = 5). Data analyzed by ANOVA with Tukey’s HSD (*p* < 0.05); homogeneity of variance confirmed via Levene’s test.

## Data Availability

Data are contained within the article and Supplementary Materials.

## Supplementary Materials

The following supporting information can be downloaded at: https://www.mdpi.com/article/10.3390/plants14213246/s1. Table S1: Raw data for the quantification of GA content, lignin precursors, intracellular H_2_O_2_ flux, and detailed concentration gradients and sampling time points for all treatment groups.
