# Supplementary material for: Unveiling the GA4-Ferulic Acid Regulatory Axis: Redox-Mediated Suberization Governs Adventitious Rooting Recalcitrance in Pinus massoniana
Source: Plants (Basel). 2025 Oct 23;14(21):3246. doi: 10.3390/plants14213246 (PMC12609085; doi:10.3390/plants14213246)
Supplement: Supplementary file 1 [file plants-14-03246-s001.zip › Table S1.pdf]

Raw data for GA content (ng/g)

Kind of GA	Treatment	Rep-1	Rep-2	Rep-3	Rep-4	Rep-5	Average
GA1	CT-0d	0	0	0	0	0	**0**
	CT-10d	0	0	0	0	0	**0**
	CT-20d	0.8	1	0.9	0.9	1	**0.92**
	CT-35d	1.7	2.1	1.8	1.9	2	**1.9**
	PBZ-0d	0.1	0.3	0.2	0.2	0.3	**0.22**
	PBZ-10d	0.5	0.7	0.6	0.6	0.7	**0.62**
	PBZ-20d	1.3	1.6	1.4	1.4	1.5	**1.44**
	PBZ-35d	1.5	1.8	1.6	1.6	1.7	**1.64**
GA15	CT-0d	1.2	1.4	1.3	1.3	1.4	**1.32**
	CT-10d	0	0	0	0	0	**0**
	CT-20d	0	0	0	0	0	**0**
	CT-35d	0	0	0	0	0	**0**
	PBZ-0d	0	0	0	0	0	**0**
	PBZ-10d	0	0	0	0	0	**0**
	PBZ-20d	0	0.2	0.1	0.1	0.1	**0.1**
	PBZ-35d	0.4	0.6	0.5	0.5	0.6	**0.52**
GA19	CT-0d	0	0	0	0	0	**0**
	CT-10d	0	0	0	0	0	**0**
	CT-20d	0.9	1.1	1	1	1.1	**1.02**
	CT-35d	2.7	3.1	2.8	2.9	3	**2.9**
	PBZ-0d	0	0	0	0	0	**0**
	PBZ-10d	0	0	0	0	0	**0**
	PBZ-20d	0	0	0	0	0	**0**
	PBZ-35d	0	0	0	0	0	**0**
GA4	CT-0d	0.1	0.5	0.2	0.3	0.5	**0.32**
	CT-10d	0.7	1.9	1	1.3	0.4	**1.06**
	CT-20d	0.6	2	0.9	1.3	1.7	**1.3**
	CT-35d	0.9	1.7	1.1	1.3	1.5	**1.3**
	PBZ-0d	0.2	0.8	0.3	0.5	0.7	**0.5**
	PBZ-10d	2.5	2.9	2.6	2.7	2.8	**2.7**
	PBZ-20d	10.3	11.1	10.5	10.7	10.9	**10.7**
	PBZ-35d	2.2	3	2.4	2.6	2.8	**2.6**
GA9	CT-0d	75.2	77	75.8	76.1	76.5	**76.12**
	CT-10d	34.5	37.1	35.2	35.8	36.4	**35.8**
	CT-20d	73.9	75.4	74.3	74.6	74.9	**74.62**
	CT-35d	56.6	57.8	56.9	57.2	57.5	**57.2**
	PBZ-0d	65	67.2	65.7	66.1	66.6	**66.12**
	PBZ-10d	4.1	6.7	4.7	5.4	6	**5.38**
	PBZ-20d	8	9.4	8.4	8.7	9	**8.7**
	PBZ-35d	50.4	51.6	50.7	51.0	51.3	**51.0**
GA20	CT-0d	0	0	0	0	0	**0**
	CT-10d	0	0	0	0	0	**0**
	CT-20d	0	0	0	0	0	**0**
	CT-35d	0	0	0	0	0	**0**

Kind of GA	Treatment	Rep-1	Rep-2	Rep-3	Rep-4	Rep-5	Average
	PBZ-0d	0.1	0.3	0.2	0.2	0.3	**0.22**
	PBZ-10d	0.3	0.5	0.4	0.4	0.5	**0.42**
	PBZ-20d	0.2	0.4	0.3	0.3	0.4	**0.32**
	PBZ-35d	0	0.2	0.1	0.1	0.2	**0.12**
GA29	CT-0d	0.1	0.3	0.2	0.2	0.3	**0.22**
	CT-10d	0	0.2	0.1	0.1	0.2	**0.12**
	CT-20d	0	0	0	0	0	**0**
	CT-35d	0	0.2	0.1	0.1	0.2	**0.12**
	PBZ-0d	0	0	0	0	0	**0**
	PBZ-10d	0	0	0	0	0	**0**
	PBZ-20d	0	0	0	0	0	**0**
	PBZ-35d	0	0	0	0	0	**0**
GA3	CT-0d	4.4	4.8	4.5	4.6	4.7	**4.6**
	CT-10d	12.3	12.9	12.4	12.6	12.8	**12.6**
	CT-20d	3	3.2	3.1	3.1	3.2	**3.12**
	CT-35d	3.3	3.7	3.4	3.5	3.6	**3.5**
	PBZ-0d	5.3	5.7	5.4	5.5	5.6	**5.5**
	PBZ-10d	1.3	1.7	1.4	1.5	1.6	**1.5**
	PBZ-20d	3.3	3.7	3.4	3.5	3.6	**3.5**
	PBZ-35d	2.3	2.7	2.4	2.5	2.6	**2.5**
GA5	CT-0d	0.9	1.1	1	1	1.1	**1.02**
	CT-10d	1	1.2	1.1	1.1	1.2	**1.12**
	CT-20d	0.7	0.9	0.8	0.8	0.9	**0.82**
	CT-35d	0.9	1.1	1	1	1.1	**1.02**
	PBZ-0d	0.7	0.9	0.8	0.8	0.9	**0.82**
	PBZ-10d	0.3	0.5	0.4	0.4	0.5	**0.42**
	PBZ-20d	0.4	0.6	0.5	0.5	0.6	**0.52**
	PBZ-35d	0.2	0.4	0.3	0.3	0.4	**0.32**
GA51	CT-0d	1.1	1.3	1.2	1.2	1.3	**1.22**
	CT-10d	3	3.4	3.1	3.2	3.3	**3.2**
	CT-20d	2	2.2	2.1	2.1	2.2	**2.12**
	CT-35d	5.2	5.6	5.3	5.4	5.5	**5.4**
	PBZ-0d	1.1	1.3	1.2	1.2	1.3	**1.22**
	PBZ-10d	1.4	1.6	1.5	1.5	1.6	**1.52**
	PBZ-20d	1.4	1.6	1.5	1.5	1.6	**1.52**
	PBZ-35d	0.4	0.6	0.5	0.5	0.6	**0.52**
GA6	CT-0d	0	0	0	0	0	**0**
	CT-10d	0.5	0.7	0.6	0.6	0.7	**0.62**
	CT-20d	0.3	0.5	0.4	0.4	0.5	**0.42**
	CT-35d	0.2	0.4	0.3	0.3	0.4	**0.32**
	PBZ-0d	5.7	6.1	5.8	5.9	6	**5.9**
	PBZ-10d	10.5	11.0	10.6	10.8	10.9	**10.76**
	PBZ-20d	8.7	9.1	8.8	8.9	9	**8.9**
	PBZ-35d	1.8	2.2	1.9	2	2.1	**2**
GA7	CT-0d	0	0	0	0	0	**0**
	CT-10d	0	0	0	0	0	**0**

Kind of GA	Treatment	Rep-1	Rep-2	Rep-3	Rep-4	Rep-5	Average
	CT-20d	0	0.2	0.1	0.1	0.2	**0.12**
	CT-35d	0	0	0	0	0	**0**
	PBZ-0d	0	0.2	0.1	0.1	0.2	**0.12**
	PBZ-10d	0.2	0.4	0.3	0.3	0.4	**0.32**
	PBZ-20d	1	1.2	1.1	1.1	1.2	**1.12**
	PBZ-35d	0.5	0.7	0.6	0.6	0.7	**0.62**
GA8	CT-0d	0	0	0	0	0	**0**
	CT-10d	0	0	0	0	0	**0**
	CT-20d	0	0	0	0	0	**0**
	CT-35d	0.9	1.1	1	1	1.1	**1.02**
	PBZ-0d	0	0	0	0	0	**0**
	PBZ-10d	0	0	0	0	0	**0**
	PBZ-20d	0	0	0	0	0	**0**
	PBZ-35d	0	0	0	0	0	**0**
Total GAs	CT-0d	83.9	85.5	84.3	84.7	85.1	**84.7**
	CT-10d	53.8	55.6	54.3	54.7	55.1	**54.7**
	CT-20d	82	86.4	83.1	84.2	85.3	**84.2**
	CT-35d	70.8	78.4	72.7	74.6	76.5	**74.6**
	PBZ-0d	78.2	82.8	79.4	80.5	81.6	**80.5**
	PBZ-10d	22.1	25.1	22.9	23.6	24.3	**23.6**
	PBZ-20d	33.1	40.3	34.9	36.7	38.5	**36.7**
	PBZ-35d	59.5	63.9	60.6	61.7	62.8	**61.7**

Raw data for GA_3OX activity (U/L)

Treatment	0d-rep1	0d-rep2	0d-rep3	0d-rep4	0d-rep5	**Average**	**SD**
CT	14.1	17.2	15.6	16.3	14.8	**15.6**	**1.2**
PBZ	12.5	17.4	14.9	16.1	13.6	**14.9**	**1.9**
Treatment	10d-rep1	10d-rep2	10d-rep3	10d-rep4	10d-rep5	**Average**	**SD**
CT	52.3	46.5	48.6	50.7	44.9	**48.6**	**3.0**
PBZ	82.9	76.9	78.9	80.8	74.8	**78.86**	**3.2**
Treatment	20d-rep1	20d-rep2	20d-rep3	20d-rep4	20d-rep5	**Average**	**SD**
CT	48.9	47	47.8	46.1	49.9	**47.94**	**1.5**
PBZ	151.3	146.2	148.6	143.8	153.4	**148.66**	**3.8**
Treatment	35d-rep1	35d-rep2	35d-rep3	35d-rep4	35d-rep5	**Average**	**SD**
CT	51.1	47.9	49.5	46.8	52.3	**49.52**	**2.2**
PBZ	46.8	42.3	44.6	40.1	48.9	**44.54**	**3.5**

Raw data for Lignin precursors under the control treatment

Compounds	CT-0d (Rep1)	CT-0d (Rep2)	CT-0d (Rep3)	CT-0d (Rep4)	CT-0d (Rep5)	**Average**
L-Phenylalanine	3.42E+06	3.91E+06	3.62E+06	3.85E+06	3.40E+06	**3.64E+06**
p-Coumaric acid	7.90E+04	8.80E+04	8.50E+04	9.30E+04	8.20E+04	**8.54E+04**
Caffeic acid	1.82E+05	2.54E+05	2.38E+05	2.96E+05	2.21E+05	**2.38E+05**
Ferulic acid	5.20E+04	5.90E+04	5.70E+04	6.30E+04	5.40E+04	**5.70E+04**
p-Coumaryl alcohol	5.21E+05	6.05E+05	5.76E+05	6.39E+05	5.43E+05	**5.77E+05**
coniferaldehyde	9.25E+05	1.06E+06	1.01E+06	1.10E+06	9.57E+05	**1.01E+06**
Caffeyl alcohol	5.25E+06	5.91E+06	5.72E+06	6.30E+06	5.43E+06	**5.72E+06**
Cinnamic acid	1.11E+06	1.23E+06	1.19E+06	1.29E+06	1.14E+06	**1.19E+06**
Caffeyl alcohol/FA	1.01E+02	1.00E+02	1.00E+02	1.00E+02	1.01E+02	**1.00E+02**
Compounds	CT-10d (Rep1)	CT-10d (Rep2)	CT-10d (Rep3)	CT-10d (Rep4)	CT-10d (Rep5)	**Average**
L-Phenylalanine	6.06E+06	5.99E+06	5.71E+06	5.37E+06	5.41E+06	**5.71E+06**
p-Coumaric acid	1.06E+05	9.80E+04	9.60E+04	8.80E+04	9.30E+04	**9.62E+04**
Caffeic acid	4.12E+05	3.58E+05	3.26E+05	2.59E+05	2.75E+05	**3.26E+05**
Ferulic acid	6.50E+04	6.20E+04	6.10E+04	5.80E+04	6.00E+04	**6.12E+04**
p-Coumaryl alcohol	3.89E+05	3.65E+05	3.60E+05	3.38E+05	3.47E+05	**3.60E+05**
coniferaldehyde	7.10E+05	6.85E+05	6.37E+05	5.79E+05	5.96E+05	**6.41E+05**
Caffeyl alcohol	9.67E+06	9.46E+06	9.32E+06	9.03E+06	9.15E+06	**9.32E+06**
Cinnamic acid	9.38E+05	8.95E+05	8.65E+05	8.09E+05	8.28E+05	**8.67E+05**
Caffeyl alcohol/FA	1.49E+02	1.53E+02	1.53E+02	1.56E+02	1.52E+02	**1.52E+02**
Compounds	CT-20d (Rep1)	CT-20d (Rep2)	CT-20d (Rep3)	CT-20d (Rep4)	CT-20d (Rep5)	**Average**
L-Phenylalanine	4.69E+06	3.90E+06	4.23E+06	3.81E+06	4.55E+06	**4.23E+06**
p-Coumaric acid	1.89E+05	1.68E+05	1.76E+05	1.65E+05	1.74E+05	**1.74E+05**
Caffeic acid	1.13E+06	1.05E+06	1.07E+06	1.02E+06	1.08E+06	**1.07E+06**
Ferulic acid	1.27E+05	1.19E+05	1.21E+05	1.16E+05	1.22E+05	**1.21E+05**
p-Coumaryl alcohol	6.10E+05	5.29E+05	5.56E+05	5.12E+05	5.83E+05	**5.58E+05**
coniferaldehyde	1.02E+06	9.01E+05	9.39E+05	8.73E+05	9.85E+05	**9.44E+05**
Caffeyl alcohol	6.51E+06	5.61E+06	5.96E+06	5.46E+06	6.26E+06	**5.96E+06**
Cinnamic acid	5.54E+05	4.88E+05	5.11E+05	4.75E+05	5.32E+05	**5.12E+05**
Caffeyl alcohol/FA	5.13E+01	4.71E+01	4.93E+01	4.71E+01	5.13E+01	**4.92E+01**
Compounds	CT-35d (Rep1)	CT-35d (Rep2)	CT-35d (Rep3)	CT-35d (Rep4)	CT-35d (Rep5)	**Average**
L-Phenylalanine	4.45E+06	5.30E+06	4.89E+06	5.42E+06	4.39E+06	**4.89E+06**
p-Coumaric acid	1.75E+05	2.03E+05	1.97E+05	2.18E+05	1.86E+05	**1.96E+05**
Caffeic acid	3.11E+06	3.19E+06	3.15E+06	3.20E+06	3.12E+06	**3.15E+06**
Ferulic acid	2.16E+05	2.24E+05	2.22E+05	2.29E+05	2.19E+05	**2.22E+05**
p-Coumaryl alcohol	5.21E+05	5.92E+05	5.65E+05	6.17E+05	5.38E+05	**5.67E+05**
coniferaldehyde	1.80E+06	1.98E+06	1.92E+06	2.06E+06	1.85E+06	**1.92E+06**
Caffeyl alcohol	5.50E+06	6.46E+06	6.08E+06	6.73E+06	5.69E+06	**6.09E+06**
Cinnamic acid	8.43E+05	9.28E+05	9.01E+05	9.65E+05	8.65E+05	**9.00E+05**
Caffeyl alcohol/FA	2.55E+01	2.88E+01	2.74E+01	2.94E+01	2.60E+01	**2.74E+01**

Raw data for Lignin precursors under GA_4 treatment

Compounds	GA4-0d (Rep1)	GA4-0d (Rep2)	GA4-0d (Rep3)	GA4-0d (Rep4)	GA4-0d (Rep5)	**Average**
L-Phenylalanine	3.56E+06	3.46E+06	3.50E+06	3.39E+06	3.59E+06	**3.50E+06**
p-Coumaric acid	9.40E+04	8.80E+04	9.00E+04	8.30E+04	9.60E+04	**9.02E+04**
Caffeic acid	2.49E+05	2.25E+05	2.35E+05	2.08E+05	2.58E+05	**2.35E+05**
Ferulic acid	5.50E+04	5.30E+04	5.40E+04	5.10E+04	5.60E+04	**5.38E+04**
p-Coumaryl alcohol	6.47E+05	5.92E+05	6.18E+05	5.63E+05	6.71E+05	**6.18E+05**
coniferaldehyde	9.84E+05	9.28E+05	9.54E+05	8.97E+05	1.01E+06	**9.54E+05**
Caffeyl alcohol	6.19E+06	5.95E+06	6.06E+06	5.80E+06	6.30E+06	**6.06E+06**
Cinnamic acid	2.62E+06	2.50E+06	2.56E+06	2.45E+06	2.68E+06	**2.56E+06**
Caffeyl alcohol/FA	1.13E+02	1.12E+02	1.12E+02	1.14E+02	1.12E+02	**1.13E+02**
Compounds	GA4-10d (Rep1)	GA4-10d (Rep2)	GA4-10d (Rep3)	GA4-10d (Rep4)	GA4-10d (Rep5)	**Average**
L-Phenylalanine	5.12E+06	5.33E+06	5.44E+06	5.60E+06	5.67E+06	**5.43E+06**
p-Coumaric acid	9.40E+04	9.80E+04	1.01E+05	1.05E+05	1.08E+05	**1.01E+05**
Caffeic acid	2.95E+05	3.52E+05	3.99E+05	4.48E+05	4.89E+05	**3.97E+05**
Ferulic acid	6.20E+04	6.40E+04	6.60E+04	6.80E+04	6.90E+04	**6.58E+04**
p-Coumaryl alcohol	2.63E+05	2.76E+05	2.82E+05	2.91E+05	2.98E+05	**2.82E+05**
coniferaldehyde	7.37E+05	7.82E+05	8.14E+05	8.53E+05	8.86E+05	**8.14E+05**
Caffeyl alcohol	5.14E+06	5.31E+06	5.47E+06	5.65E+06	5.79E+06	**5.47E+06**
Cinnamic acid	2.88E+06	3.02E+06	3.13E+06	3.26E+06	3.36E+06	**3.13E+06**
Caffeyl alcohol/FA	8.28E+01	8.29E+01	8.29E+01	8.31E+01	8.39E+01	**8.31E+01**
Compounds	GA4-20d (Rep1)	GA4-20d (Rep2)	GA4-20d (Rep3)	GA4-20d (Rep4)	GA4-20d (Rep5)	**Average**
L-Phenylalanine	4.33E+06	4.63E+06	4.48E+06	4.56E+06	4.43E+06	**4.49E+06**
p-Coumaric acid	2.68E+05	3.00E+05	2.85E+05	2.93E+05	2.79E+05	**2.85E+05**
Caffeic acid	4.02E+06	4.30E+06	4.16E+06	4.24E+06	4.10E+06	**4.16E+06**
Ferulic acid	2.82E+05	2.97E+05	2.90E+05	2.94E+05	2.87E+05	**2.90E+05**
p-Coumaryl alcohol	5.32E+05	6.09E+05	5.72E+05	5.93E+05	5.56E+05	**5.72E+05**
coniferaldehyde	1.02E+06	1.26E+06	1.14E+06	1.21E+06	1.08E+06	**1.14E+06**
Caffeyl alcohol	2.48E+06	2.95E+06	2.72E+06	2.85E+06	2.61E+06	**2.72E+06**
Cinnamic acid	1.17E+06	1.36E+06	1.27E+06	1.32E+06	1.23E+06	**1.27E+06**
Caffeyl alcohol/FA	8.79E+00	9.93E+00	9.38E+00	9.69E+00	9.09E+00	**9.38E+00**
Compounds	GA4-35d (Rep1)	GA4-35d (Rep2)	GA4-35d (Rep3)	GA4-35d (Rep4)	GA4-35d (Rep5)	**Average**
L-Phenylalanine	4.82E+06	4.49E+06	4.63E+06	4.33E+06	4.90E+06	**4.63E+06**
p-Coumaric acid	1.32E+05	1.23E+05	1.27E+05	1.18E+05	1.36E+05	**1.27E+05**
Caffeic acid	4.40E+06	4.32E+06	4.35E+06	4.27E+06	4.43E+06	**4.35E+06**
Ferulic acid	2.92E+05	2.68E+05	2.79E+05	2.53E+05	3.03E+05	**2.79E+05**
p-Coumaryl alcohol	4.98E+05	4.59E+05	4.76E+05	4.38E+05	5.13E+05	**4.77E+05**
coniferaldehyde	2.73E+06	2.53E+06	2.62E+06	2.42E+06	2.81E+06	**2.62E+06**
Caffeyl alcohol	2.25E+06	1.81E+06	2.01E+06	1.59E+06	2.40E+06	**2.01E+06**
Cinnamic acid	4.94E+05	4.32E+05	4.61E+05	3.97E+05	5.19E+05	**4.61E+05**
Caffeyl alcohol/FA	7.70E+00	6.75E+00	7.20E+00	6.28E+00	7.91E+00	**7.17E+00**

Raw data for H_2O_2 flux

Measuring time (Second)	CK-0d	CK-20d	F1-20d	F2-20d	F3-20d
0	6.6622	-4.4794	9.1030	1.3838	-1.5734
30	1.3066	-7.0046	-7.6080	6.5970	2.0934
60	-0.3736	-14.3724	-4.9676	-2.4296	0.3604
90	-5.3038	-3.8836	2.1940	-4.1484	-0.5228
120	7.6434	-14.4116	1.8420	-5.8084	13.5360
150	0.2592	-11.1064	-11.6526	1.1352	2.8048
180	-1.7676	5.6730	8.4314	-3.0130	0.9078
210	1.3801	-19.5228	-9.8722	-2.4062	6.0318
240	-2.9316	-8.1296	-8.9414	0.3984	-2.5546
270	2.8416	1.0172	-2.0642	-0.9528	-3.7662
300	-7.8944	-27.1123	-14.6036	-1.9162	-0.0646
330	-8.7130	-6.8110	-7.2566	-5.0606	-2.1656
360	-2.8128	-12.1178	8.3094	7.4856	1.9430
390	-6.0734	-6.5440	2.8396	-0.2956	1.0964
420	-7.1478	-14.3532	-9.1724	-3.4378	3.4344
450	2.0318	3.0992	4.0798	0.7411	-2.1162
480	1.8666	-4.1392	1.1830	6.5584	5.8078
510	6.3614	5.0368	3.5224	-3.9138	3.9674
540	6.2658	-7.7292	-1.9701	2.1680	0.0230
570	-6.0734	-6.5440	2.8396	1.1342	-2.5290
600	7.3482	-2.5512	-0.6238	-4.0444	-1.5704
**Average**	**-0.2440**	**-7.4279**	**-1.6375**	**-0.4679**	**1.1973**
**SD**	**5.3181**	**8.0061**	**6.9850**	**3.8299**	**3.9398**

Detailed concentration gradients and sampling time points for all treatment groups

Treatment Group	NAA ($mg \cdot L^{-1}$)	PBZ ($mg \cdot L^{-1}$)	GA_4 ($mg \cdot L^{-1}$)	FA ($mg \cdot L^{-1}$)	Sampling Time Points (Days)
Control	200	0	0	0	0, 10, 20, 35
PBZ-Low	200	50	0	0	0, 10, 20, 35
PBZ-Optimal	200	100	0	0	0, 10, 20, 35
PBZ-High	200	200	0	0	0, 10, 20, 35
GA_4-Low	200	0	50	0	0, 10, 20, 35
GA_4-Med	200	0	100	0	0, 10, 20, 35
FA-Optimal	200	0	0	200	0, 10, 20, 35
FA-High	200	0	0	600/1000	0, 10, 20, 35
PBZ+FA	200	100	0	200	0, 10, 20, 35
